# Gender-Pain Questionnaire: Internal Validation of a Scale for Assessing the Influence of Chronic Pain Experience on Gender Identity and Roles

**DOI:** 10.3390/clinpract15100176

**Published:** 2025-09-25

**Authors:** Ana M. Peiró, Noelia Serrano-Gadea, Daniel García-Torres, María Teresa Ruiz-Cantero, Virtudes Pérez-Jover

**Affiliations:** 1 Clinical Pharmacology, Toxicology and Chemical Safety Unit, Institute of Bioengineering, Miguel Hernández University, 03202 Elche, Alicante, Spain; nserrano@umh.es; 2 Neuropharmacology Applied to Pain (NED), Clinical Pharmacology Unit, Dr. Balmis General University Hospital, Alicante Institute for Health and Biomedical Research (ISABIAL), 03010 Alicante, Spain; 3 ATENEA Research Foundation for the Promotion of Health and Biomedical Research of Valencia Region, FISABIO, 46020 Valencia, Spain; daniel.garciat@umh.es; 4 Public Health Research Group, University of Alicante, 03690 San Vicente del Raspeig, Alicante, Spain; cantero@ua.es; 5 Biomedical Research Centre in Epidemiology and Public Health Network (CIBERESP), 28029 Madrid, Spain; 6 Health Psychology Department, Miguel Hernández University, 03202 Elche, Alicante, Spain; v.perez@umh.es; 7 Research in Hospital Psychology, Alicante Institute for Health and Biomedical Research (ISABIAL), 03010 Alicante, Spain

**Keywords:** gender, chronic pain, identity, relationships, work, gender roles, reliability, validity

## Abstract

**Background/Objectives:** Gender (roles as household load and job strain, and identity) represent an effect modifier of the interference between pain experience and sex because it is different between men and women. This study validates a new scale developed to assess how life functioning is impacted by Chronic Non-Cancer Pain (CNCP) due to gender. **Methods:** A total of 193 Spanish ambulatory CNCP patients (60 [51–73] years old, 69.4% women, 31% retired) were interviewed. Exploratory Factor Analysis (EFA) yielded 3-factor structure: Gender Self-identity, Roles, and Chronic Pain Impact on Social, Familial, Work and Sexual Life. **Results:** The Gender-Pain Questionnaire, with the presented factor structure, is an evaluation instrument with enough reliability and internal validity for CNCP patients. **Conclusions:** This study presents the psychometric properties of a scale for assessing the interference of CNCP patients’ experience on gender and how it affects their daily life activities, relationships and self-identity. It represents the first original questionnaire known in Spanish language to date. This measure could potentially help researchers and clinicians to obtain gender key information to design appropriate and equity healthcare interventions.

## 1. Introduction

The Institute of Medicine of the U.S. recognized, more than two decades ago, that biological sex is a determining factor in health outcomes throughout our lifetime as well as gender (social and cultural behaviours) [1,2]. The Canadian Institutes of Health Research [3] and the European Commission [4] have endorsed integrating sex and gender (usually as male/female binaries) into health research, and the U.S. The National Institutes of Health has mandated it, too [5]. However, still to this day, these two concepts are often confused in the literature [6,7,8,9], and there is also a lack of quantitative tools to analyze the influence of gender on health outcomes, including in the field of pain management.

The term “sex” refers to biological differences between men and women, specifically reproductive organs and their functions. The term “gender” is a multidimensional concept that comprises different aspects such as gender identity (how an individual sees themselves and relates to masculinity and femininity), roles (behavioural customs applied to sexes in societies that have an influence in their daily lives and experiences), and relationships (how gender shapes social interactions) [10]. It is based on cultural norms, and it denotes the social context in which we live [10,11].

Nowadays, gender is regarded as a complex concept based on multiple areas of people’s social life [12]. In other words, the gender concept allows us to discover how men and women internalize cultural norms and socially direct themselves [10]. One aspect of gender are roles that have been usually described as a dual trait (femininity/masculinity) and later defined as a spectrum [9].

The term femininity is associated with expressivity, and masculinity with instrumental orientation (jobs) and leadership abilities [13]. Thus, the productive role refers to paid workers meanwhile the reproductive role to individuals overseeing domestic tasks [14]. The latter is usually applied to females that are in charge of childcare, parenting assistance, cooking, cleaning, i.e., if women have a drug problem they may be questioned, for example, to be suitable as a “mother” [15]. Previous studies have revealed clear differences in pain tolerance based on gender roles [16] and the aforementioned gender stereotypes influence doctor-patient communication [17]. In a similar way, gender identity is attributed to masculine characteristics such as assertiveness or aggressiveness, meanwhile to feminine characteristics such as affection and sympathy [18].

To understand why gender could be a cause of differences in psychosocial health, social inequalities must be understood [19,20]. For example, women tend to report greater receipt of prescriptions for anxiolytics, sedatives or hypnotics [21] due to the assumption of higher emotional issues. Meanwhile, women report feeling more pain than men, their pain is often underdiagnosed and undertreated [22]. Moreover, evidence suggests that the presence of chronic pain does not allow patients to achieve the ultimate standards of being male or female in our societies due to self-identity [23]. For example, Samulowitz et al. demonstrated a variety of gender bias in pain treatment as part of the patient-professional encounter and the professional’s treatment decisions. They also discussed how gendered norms are consolidated by hegemonic masculinity and andronormativity [24]. We will need to develop new questionnaires in order to determine whether healthcare professionals use stereotypic pain-related attributions, and how that may influence clinical pain management [25].

Briefly, in the present day, 1/we lack a tool that assesses how pain interferes with patients’ daily life based on gender (self-identity and roles); 2/we need to understand differences between men and women. Therefore, our aim was to validate a 15-question questionnaire to determine patients’ perception of pain’s impact on different areas of their life due to gender.

## 2. Materials and Methods

This study’s protocol adhered to the three phases of scale creation outlined by Boateng et al. (i.e., item development, scale development, and scale evaluation) [26]. It also followed the standards and guidelines for validation practices summarized by Chan [27], as well as the recommendations from the COnsensus-based Standards for the selection of health status Measurement INstruments (COSMIN) [28].

### 2.1. Item Development

The 15-items gender questionnaire was designed based on a previous cross-sectional study with spondyloarthritis patients, whose sources of information were semi-structured patient interviews [29,30]. The main objective was to illustrate how the gender perspective (roles and identity) can contribute to contextualizing the differences by sex of functional alterations (relationships and daily life). The experts who designed the questions came from the Public Health Research Group, University of Alicante (Spain) and Department of Rheumatology, Alicante University General Hospital, Alicante (Spain).

Here, the validation is applied to Chronic Non-Cancer Pain (CNCP) patients from the same health area. In a similar way, three trained interviewers conducted face-to-face interviews that lasted 30–45 min. Quantitative information was obtained by collecting the answers (yes/no) to the 15 questions about gender roles or gender identity. Here, questions 2–6 were related to gender identity (female/male), while gender roles were related to questions 7 (work), 9 (domestic responsibilities), 11–12 (partner relationships) and 13 (family) [31]. Self-reported gender roles were reproductive role (childbearing and caring for children) which refers to unpaid domestic tasks to maintain homes (cooking, fetching water, cleaning, washing clothes and similar) and productive role, which is work performed to produce goods and services for consumption or trade [14]. These gender roles are associated in society with men and women, respectively, in a stereotypical manner [32].

### 2.2. Item Scoring

The questions’ responses were recorded on a dichotomous scale (0 = No, 1 = Yes). This method was used and tested to capture patients’ perspectives. Total scores were calculated by summing the individual item scores. Higher scores indicate greater self-perception of pain.

### 2.3. Content and Face Validity

The criteria for inclusion were CNCP adults, outpatients from our centre who regularly come to their clinical visits, and willing to participate in a study. In addition, the questionnaire was given to a clinical psychologist and three pain researchers (one individual with a PhD in medicine, two with a PhD in pharmacy) who were familiar with the concept under investigation and instrumentation. All of them were required to evaluate the items with respect to appropriate wording and grammar, understandability, and to mention their suggestions, if any, next to each item.

The first 50 CNCP patients took part in this component of the study. The participants were required to evaluate the items with respect to problems, ambiguity, relativity, proper terms and grammar, and understandability. A short training on patient interviews was provided to the researchers who had more experience with quantitative methods. After that, general instructions related to how to interview the patients were reached through consensus. In addition, a meeting was held with a group of epidemiologists who are experts in qualitative research to preliminarily assess the quality of the data.

### 2.4. Internal Validity Study

**Figure 1:**
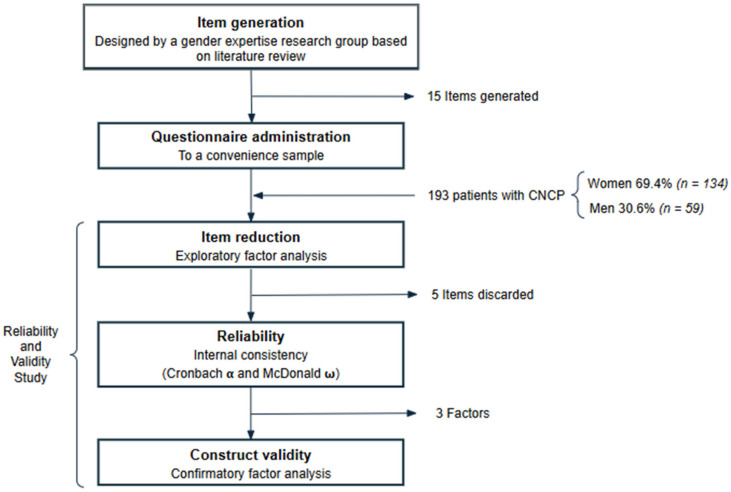
Flow diagram of the Gender-Pain Questionnaire validation process.

**Figure 2:**
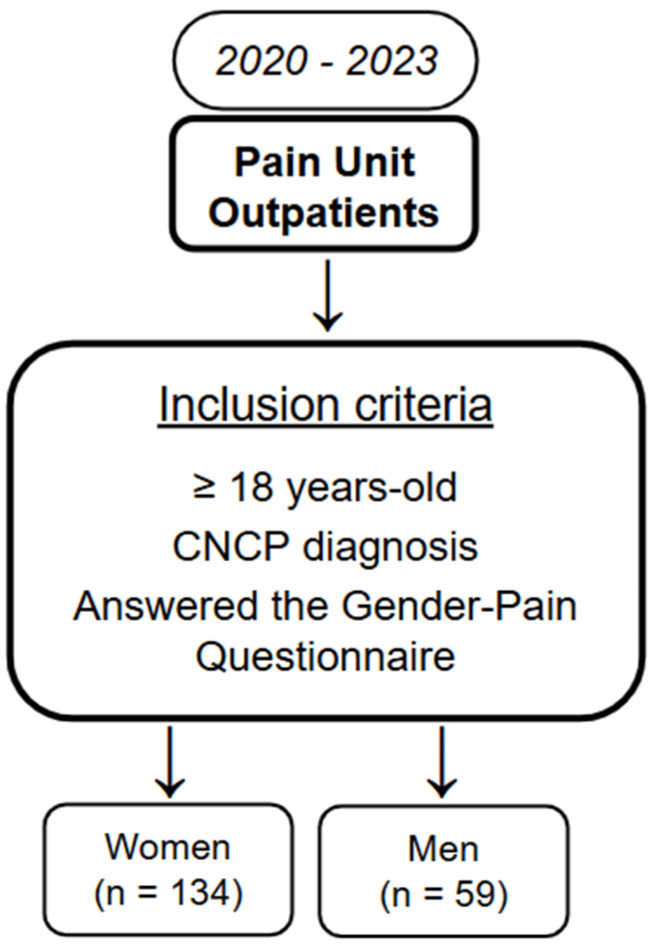
Flow chart of the patients included in the study according to sex.

A paper-based validation study was performed from September 2020 to November 2023 at the PU of the Alicante Health Department of the Dr. Balmis General University Hospital in Spain to gather data and evaluate the measurement and psychometric properties of the Questionnaire (Figure 1). It included 193 patients with CNCP (Figure 2) that provided quantitative and qualitative information. The inclusion criteria were adults aged ≥ 18 years with CNCP who signed an informed consent. The exclusion criteria were patients with oncologic pain or that did not meet IASP’s (International Association for the Study of Pain) diagnostic algorithm, diagnosis of terminal illness with a survival rate of less than six-month prognosis, and/or any psychiatric disorder that could interfere with properly performing this study were excluded. The study did not incorporate chronic pain conditions of unknown pathophysiology, such as fibromyalgia or neuropathic pain conditions (painful polyneuropathy, postherpetic neuralgia, trigeminal neuralgia, and post-stroke pain) [33].

Sample size was determined using the formula for finite populations, considering the most recent prevalence data of chronic pain in the Valencian Community (26.1%) [34]. A minimum sample size was established based on the recommendation of including at least 10 participants per item. Given that the original questionnaire consists of 15 items, a sample size of at least 150 participants was determined to ensure adequate representation and enable robust statistical analyses of validity and reliability. This sample size is sufficient for conducting factor analyses to assess the dimensional structure of the questionnaire, as well as for calculating internal consistency indicators, such as Cronbach’s *α*, thereby supporting a thorough validation of the instrument.

A consecutive sampling method was used with outpatients (Figure 2). The researchers prepared the questionnaires and informed consents. When patients met the inclusion criteria, they were informed by the PU healthcare team about the purpose of the study. Then, any interested individuals were asked by the research staff to sign an informed consent and all variables were collected. All the patients were self-reported as cis (“female” or “male”: the sample included no non-binary person) and a consecutive number participant identifier was assigned.

#### 2.4.1. Item Reduction

Exploratory factor analysis (EFA) using the principal component method was conducted to evaluate the latent structure of the scales. A Varimax rotation was applied to achieve clearer results and facilitate the interpretation of factors. Items with factor loadings below 0.50 or with significant cross-loadings were excluded from the model. The analysis was performed using IBM SPSS Statistics for Windows, Version 25.0. Additionally, the remaining items were reviewed by a multidisciplinary research team composed of medical doctors, psychologists, experts in mixed-methods research, and patients. This collaborative process ensured that the retained items were relevant and comprehensible for the target population of the instrument.

#### 2.4.2. Internal Consistency

To assess the internal consistency of the scale, both Cronbach’s *α* and McDonald’s *ω* were calculated. Cronbach’s *α* provides an estimate of how well the items within each factor are correlated, indicating the internal coherence of the scale. Additionally, McDonald’s *ω* was calculated as it offers a more accurate estimate of internal consistency, especially in cases where the assumption of tau-equivalence is not met. A minimum value of 0.70 was considered acceptable to indicate adequate internal consistency.

### 2.5. Ethics Statement

This study was approved by the Ethics Committee Board of the Dr. Balmis General University Hospital of Alicante (codes PI2020-047 (29 April 2020), 2020-158 (24 March 2021)). Subjects gave verbal and signed informed consent before participating in interviews. Confidentiality of all the information was guaranteed. The study was performed in accordance with the Declaration of Helsinki regarding research involving human subjects. The generated datasets are available from the corresponding author upon reasonable request.

## 3. Results

### 3.1. Participants

The sample included 193 patients (Table 1), of which 134 (69.4%) were women and 59 (30.6%) were men, with a median age of 60 [51–73] years. Regarding current employment status, 60 patients (31%) were retired, 50 (26%) had a temporary or permanent work disability, 34 (18%) were employed, 27 (14%) homemakers-only women-(20% vs. 0%, *p* = 0.006), and 12 (6%) unemployed. Ten patients (5%) did not provide employment information. The time from pain onset to the first consultation at the Pain Unit varied significantly. A total of 45 patients (23%) were seen within 3 to 12 months after the initial pain manifestation, 34 patients (18%) between 12 and 24 months, 33 patients (18%) between 24 months and 5 years, and 80 patients (41%) experienced a delay of more than 5 years before their first consultation.

### 3.2. Item Reduction

The questions (items) were grouped in different factors and the models were evaluated. Questions 5, 6, 8, 9 and 15 of the original Questionnaire (Table 2) were omitted from the final Questionnaire (Table 3) as they did not fit a model with enough reliability and validity.

### 3.3. Internal Validity

Internal consistency values were adequate when all items of the scale were considered, although the levels varied among the factors. For Factor 1 (Identity), Cronbach’s *α* was 0.71, and McDonald’s *ω* was 0.74, indicating acceptable internal consistency for this dimension. In the case of Factor 2 (Relationships), the McDonald’s *ω* value of 0.74 suggested moderate reliability, which was slightly higher than the Cronbach’s *α* of 0.68. Moreover, the removal of item 7 could enhance the reliability of this factor, potentially increasing the Cronbach’s *α* to 0.82. Factor 3 (Work) presented lower internal consistency, with Cronbach’s *α* and McDonald’s *ω* both at 0.63, indicating limited reliability for the construct as currently composed.

## 4. Discussion

This study is the first attempt in evaluating whether gender dimensions related to Identity and Gender roles, can be impacted differently due to chronic pain experience and how it affects daily life activities, relationships and self-identity. The internal validity of the Gender-Pain Questionnaire is supported by our findings: men and women were discriminated against by their measures of masculinity and femininity, respectively, due to work activity and relationships. The results provide a Pain-Gender Questionnaire of easy assessment of patients’ perception on how their pain affects different areas of their lives with good internal consistency as a measure of reliability for group comparisons. Its brevity makes it highly suitable for epidemiological research.

The use of these gender-related variables may also help us understand if gender factors play an important role as treatment-effect modifiers and would thus need to be further considered in treatment decision-making. Biological and psychosocial explanations of different expressions of pain risk being gender blind unless understood in a gender context [35]. Two main themes were extracted and further described: (1) self-identity; and (2) gender roles. The results show that each of these dimensions has a specific and potentially different impact on men and women, according to previous data [36]. These observed differences may strongly impact pain management and outcomes (i.e., return to work or afford reproductive tasks that are usually not recorded in the assessment of pain, so they go unnoticed at an economic and social level). We suggest that daily life activities (work and family considerations), relationships and self-identity are important issues in the pain management process and that differences between men and women are likely to occur. Therefore, the present questionnaire can be useful for a better understanding of pain management from a gender perspective.

Despite there being evidence on such sex and gender differences in pain management, current guidelines do not consider sex- and gender-sensitive approaches [37]. In recent decades, gender has been recognized as a determinant of health and international organizations and scientific communities recommend including the sex variable and the gender category. Gender perspective guides have been agreed upon in research and applied to the clinic sphere. To advance it is necessary to continue deepening the knowledge of the way in which gender conditions affect specific health problems. Some studies found that the typical man was perceived to be less pain sensitive, less willing to report pain, and have more pain endurance than the typical woman [25,38]. It is interesting to note that these findings have remained consistent over the past decade despite the narrowing gap between the gender roles in many arenas (e.g., athletics, work status, income). It appears that these sex-related stereotypic attributions about pain are relatively entrenched and may require direct intervention in order to be modified [25].

Moreover, the relationship between household workload and CNCP are infrequently described in the literature [39,40]. Our consistent data that only women were homemakers [41] should be further analyzed in terms of CNCP management. Furthermore, a meta-analysis of 22 studies reported a higher risk of musculoskeletal disorders among subjects with high job strain [42], linked to sleep problems [43,44], that can decrease even more their quality of life. Their causal mechanisms could be related to the interaction between physical load and chronic stress caused by psychosocial factors that could lead to dysregulation of the hypothalamic–pituitary–adrenal axis promoting a neuroinflammatory state [45,46]. Moreover, women’s gonadal hormones and genetic/epigenetics mechanisms could increase pain sensitivity and the probability of occurrence of CNCP [47]. However, due to a lack of brief assessments, the contribution of gender to CNCP gap-and vice versa-between men and women has been understudied in clinical practice [48].

Briefly, in this study, we developed and internally validated a short screening measure of gender expression using representative survey datasets of Spain. The Gender-Pain Questionnaire with the presented factor structure is an evaluation instrument with enough reliability and validity for patients’ perception of pain’s impact on their daily life activities, relationships and self-identity. Our findings support the use of gender measures, which can turn out to be more strongly predictive of CNCP than sex.

## 5. Future Perspectives/Next Steps

A confirmatory factor analysis will be conducted in a future study to evaluate the external validity of the questionnaire, presented as the mean and standard deviation (M ± SD). This analysis aims to confirm the factor structure of the scale and determine the extent to which the items grouped together as theoretically expected. Maximum likelihood extraction method will be used, and the model fit will be assessed using various fit indices (Comparative Fit Index, Tucker–Lewis Index, Root Mean Square Error of Approximation). The analysis involves examining the factor loadings of each item to ensure they are appropriately loaded onto the hypothesized factors. Items with low loadings or cross-loadings are scrutinized and evaluated for potential removal to improve the overall model fit.

## 6. Limitations

There are some limitations in this study that need to be acknowledged. Firstly, the sample was limited by patients’ similar demographics (mainly Caucasian middle-aged women) who came from a single Spaniard hospital. Secondly, the study acknowledges the lack of non-binary categories and measures only two dimensions of gender, namely self-identification and gender roles. As gender is multidimensional, any given individual may experience different configurations of gender norms, traits, and relations that may not be subsumed into a “masculine” or “feminine” score or considered “fixed” [49]. In addition, another limitation is that the Work factor showed internal consistency below the commonly accepted threshold (Cronbach’s α and McDonald’s ω = 0.63). Although retained for its clinical relevance, this subscale should be interpreted with caution and refined in future validations. Moreover, there is also the need to understand the mechanisms and pathways underlying the trends we observe, as well as how sex and gender intersect with other factors (age, socioeconomic status, employment) [41] that contribute to our health outcomes [50]. Finally, strengths of the study refer to the real-world population that comes from a diverse ambulatory clinical visit from a Hospital Pain Unit. In fact, our intervention definition is consistent providing high quality data from face-to-face clinical interviews. Moreover, this is a large and representative sample from the Spanish general population, available for comparability with other cohorts for other co-variables analysis. The present study responds to the need for self-report tools for their use in clinical and research related to gender and CNCP interference.

## Figures and Tables

**Table 1:** Sociodemographic characteristics by sex (women, men).

	Total (*n* = 193)	Women (*n* = 134)	Men (*n* = 59)	*p*-Value
Age (Med [IQR])	60 [51–73]	65 [52–75]	56 [49–66]	
**Employment status (%)**				
Active	34 (18)	23 (17)	11 (18)	0.006
Unemployed	12 (6)	8 (6)	4 (7)	
Retired	60 (31)	40 (30)	20 (34)	
Homemaker	27 (14)	27 (20)	0 ****	
Disability	50 (26)	30 (22)	20 (34)	
NA	10 (5)	6 (5)	4 (7)	-
**Diagnostic delay (%)**				
3–12 months	45 (23)	29 (21)	16 (27)	0.524
12–24 months	34 (18)	21 (16)	13 (22)	
24 months−5 years	33 (17)	24 (18)	9 (15)	
More than 5 years	80 (41)	59 (44)	21 (36)	
NA	1 (1)	1 (1)	0	-

NA: not available. **** *p* < 0.0001 when comparing women and men.

**Table 2:** Initial Gender-Pain Questionnaire to patients.

1. Has your pain changed the way you are? Yes/No. How?
2. Has the pain affected your self-esteem as a woman/man? Yes/No. How?
3. Has the pain changed your image of yourself as a man/woman? Yes/No. How?
4. Has the pain changed your masculinity or femininity? Yes/No. How?
5. Has the pain generated a conflict between what you want/can (do) and what you think your family environment asks of you as a woman/man? Yes/No. How?
6. Has the pain generated a conflict between what you want/can (do) and what the social environment asks of you as a woman/man? Yes/No. How?
7. Has the pain affected your work tasks and/or responsibilities within your work environment? Yes/No. How?
8. Did you do household chores before the diagnosis of the disease? Yes/No.
9. Has the pain affected your tasks and/or domestic responsibilities? Yes/No. How?
10. Has the pain affected your life project or your future plans? Yes/No. How?
11. Has the pain affected your relationships? Yes/No. How?
12. Has the pain affected your sexual relationships? Yes/No. How?
13. Has the pain affected your family relationships? Yes/No. How?
14. Do you think that your social, work or family position has worsened due to the pain? Yes/No. How?
15. Do you think that the experience of pain would have been different instead of a man being a woman (or vice versa)? Yes/No. How?

**Table 3:** Validated Gender-Pain Questionnaire in chronic pain experience.

**Identity**
1. Has your pain changed the way you are? Yes/No. How?
2. Has the pain affected your self-esteem as a woman/man? Yes/No. How?
3. Has the pain changed your image of yourself as a man/woman? Yes/No. How?
4. Has the pain changed your masculinity or femininity? Yes/No. How?
**Relationships**
5. Has the pain affected your relationships? Yes/No. How?
6. Has the pain affected your sexual relationships? Yes/No. How?
7. Has the pain affected your family relationships? Yes/No. How?
**Work**
8. Has the pain affected your work tasks and/or responsibilities within your work environment? Yes/No. How?
9. Has the pain affected your life project or your future plans? Yes/No. How?
10. Do you think that your social, work or family position has worsened due to the pain? Yes/No. How?

## Data Availability

The original data presented in the study are openly available in Zenodo at https://doi.org/10.5281/zenodo.14356174.

## Abbreviations

The following abbreviations are used in this manuscript:

CFA	Confirmatory Factor Analysis
CNCP	Chronic Non-Cancer Pain
COSMIN	COnsensus-based Standards for the selection of health status Measurement INstruments
EFA	Exploratory Factor Analysis
PU	Pain Unit
